# Mutant Prpf31 causes pre-mRNA splicing defects and rod photoreceptor cell degeneration in a zebrafish model for Retinitis pigmentosa

**DOI:** 10.1186/1750-1326-6-56

**Published:** 2011-07-30

**Authors:** Jun Yin, Jan Brocher, Utz Fischer, Christoph Winkler

**Affiliations:** 1 Department of Biological Sciences; National University of Singapore; 117543, Singapore; 2 Department of Biochemistry, Biocenter, University of Wuerzburg, 97074, Germany; 3 Department of Biochemistry and Molecular Medicine, School of Medicine, University of California Davis, One Shields Ave, Davis, CA 95616 USA; 4 Division of Experimental Orthopaedics, Orthopaedic University Hospital of Heidelberg, Schlierbacher Landstrasse 200, 69115 Heidelberg, Germany

**Keywords:** Retinitis pigmentosa (RP), PRPF31, AD5 mutation, SP117 mutation, haploinsufficiency, dominant-negative, rod degeneration, apoptosis, splicing defect

## Abstract

**Background:**

Retinitis pigmentosa (RP) is an inherited eye disease characterized by the progressive degeneration of rod photoreceptor cells. Mutations in pre-mRNA splicing factors including PRPF31 have been identified as cause for RP, raising the question how mutations in general factors lead to tissue specific defects.

**Results:**

We have recently shown that the zebrafish serves as an excellent model allowing the recapitulation of key events of RP. Here we use this model to investigate two pathogenic mutations in *PRPF31*, SP117 and AD5, causing the autosomal dominant form of RP. We show that SP117 leads to an unstable protein that is mislocalized to the rod cytoplasm. Importantly, its overexpression does not result in photoreceptor degeneration suggesting haploinsufficiency as the underlying cause in human RP patients carrying SP117. In contrast, overexpression of AD5 results in embryonic lethality, which can be rescued by wild-type Prpf31. Transgenic retina-specific expression of AD5 reveals that stable AD5 protein is initially localized in the nucleus but later found in the cytoplasm concurrent with progressing rod outer segment degeneration and apoptosis. Importantly, we show for the first time *in vivo *that retinal transcripts are wrongly spliced in adult transgenic retinas expressing AD5 and exhibiting increased apoptosis in rod photoreceptors.

**Conclusion:**

Our data suggest that distinct mutations in Prpf31 can lead to photoreceptor degeneration through different mechanisms, by haploinsufficiency or dominant-negative effects. Analyzing the AD5 effects in our animal model *in vivo*, our data imply that aberrant splicing of distinct retinal transcripts contributes to the observed retina defects.

## Background

Retinitis pigmentosa (RP), a general cause for blindness, is a clinically and genetically highly heterogeneous disorder affecting approximately 1.6 million people worldwide [1]. This hereditary disease is characterized by the progressive degeneration of photoreceptor cells in the retina, firstly affects rods and later results in the loss of cones. Due to the sequential loss of rods and cones, RP patients initially develop symptoms of night blindness followed by a progressive loss of the visual field from the periphery to the center until eventually complete blindness manifests [2]. Clinically, RP patients show abnormal electroretinograms (ERGs) and bone spicule-like pigment deposits in the retina after which the disease is named [2]. RP can be inherited as autosomal dominant, autosomal recessive or X-linked traits [3-5].

Currently, mutations in more than 50 genes have been associated with RP etiology [5] (also see RetNet: http://www.sph.uth.tmc.edu/RetNet/disease.htm). The majority of affected genes encodes for retina specific proteins predominantly involved in photoreception. However, surprisingly, RP mutations have also been identified in a group of housekeeping genes that are involved in pre-mRNA splicing and represent the second-largest contribution to RP after mutations in rhodopsin [6-8]. These genes include *PRPF3 *[9], *PRPF8 *[10], *PRPF31 *[11], *PAP1 *[12,13] and *SNRN200 *[14]. All these genes encode core components of the U4/U6.U5 tri-snRNP complex which constitutes a major building block of the pre-mRNA processing spliceosome [5,15]. This macromolecular machine is assembled on introns of pre-mRNAs in a step-wise fashion from five small nuclear ribonucleoproteins (snRNPs; U1, U2, U4/U6 and U5) and a multitude of accessory factors and facilitates the faithful excision of introns and the ligation of coding exons [14,16-18]. All five splicing factors involved in RP are required for the formation of the U4/U6.U5 tri-snRNP complex. PRPF31 interacts with U4 and is required during U4/U6.U5 tri-snRNP assembly and maintenance in spliceosomes [19,20].

The human *PRPF31 *gene contains 14 exons and encodes a 61 kDa protein of 499 amino acids, which is highly conserved through evolution [21]. Although *PRPF31 *is ubiquitously expressed, patients with mutant *PRPF31 *alleles only show symptoms in the retina but not other organs [22]. At present, more than 30 mutations have been identified in *PRPF31 *in RP11 linked families and sporadic cases [5,7,11,23-27]. This includes missense or nonsense substitutions, deletions as well as insertions. So far, mainly cell-culture approaches have been used to study mechanisms of *PRPF31 *mutations, e.g. in lymphoblastoid cell lines from RP patients [27-29], yeast [30], and mammalian retina cell lines [31,32]. Such cell culture approaches, however, have several limitations in recapitulating the complex *in vivo *processes during RP pathogenesis, such as necessary cell-cell interactions in a dynamic, multilayered organ. Recently, three mouse models were generated by targeting *PRPF3 *and *PRPF31 *using knock-in and knock-out approaches to explore mechanism underlying RP [8,33]. These mice unexpectedly exhibited degenerative changes in the retinal pigment epithelium (RPE), however unlike human RP patients, did not show any degeneration in the photoreceptor layer.

We have recently shown that zebrafish serves as an excellent *in vivo *model to study the consequences of *prpf31 *gene knock-down on retina function [15]. Morphants with reduced *prpf31 *expression exhibited defects in vision, photoreceptor morphology and down-regulation of retina specific transcripts. In the present study, we use this model to investigate the mechanisms by which two reported *PRPF31 *mutations, AD5 and SP117 contribute to RP pathogenesis. We show that AD5 and SP117 proteins have different stabilities and effects on embryonic survival after injection of individual mRNAs into zebrafish embryos. Both proteins show distinct subcellular localization in zebrafish photoreceptor cells and differently affect rod cell maintenance. We detected aberrantly spliced pre-mRNAs in the retinas of zebrafish overexpressing AD5 indicating dominant-negative activity of this variant. Hence, our studies provide novel insights into the pathomechanism of reported RP mutations and suggest that zebrafish can be used as a powerful animal model for efficiently validating pathogenic RP mutations.

## Results

### Generation of zebrafish Prpf31 variants mimicking human AD5 and SP117 mutations

**Figure 1:**
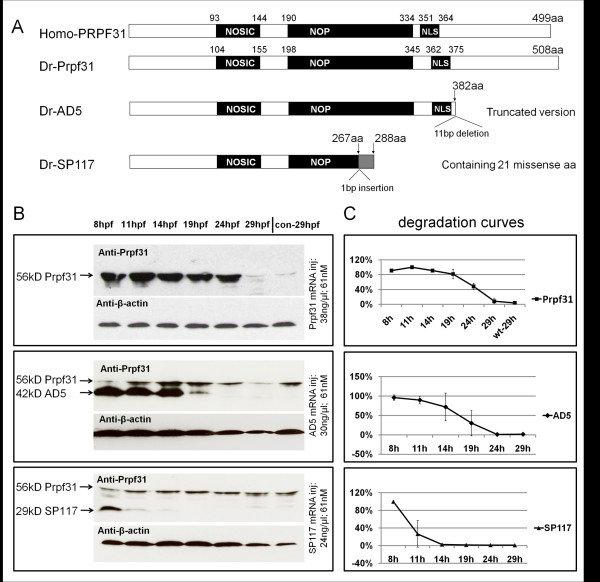
**Composition and stability of PRPF31 and its two variants**. (A) Schematic diagram of human and zebrafish PRPF31 and two mutants which mimic human AD5 and SP117 variants. Position of NOSIC and NOP domains (for details see text) and predicted nuclear localization signal (NLS) are indicated. The zebrafish AD5 variant is a truncated version (382 aa) to mimic human AD5 which has a 11 bp deletion leading to a frameshift after amino acid 371 (corresponding to zebrafish aa 382; see Additional file 1, Figure S1). SP117 mutation: A 1 bp insertion at 801/802 (in human at 769/770) leads to a frameshift after aa 267 (in human after aa 256), leading to 21 missense aa. (B) Western blot assays showing protein levels of Prpf31, AD5 and SP117 mutant variants over 29 hpf after injection of 61 nM mRNAs, respectively. "con" refers to non-injected wild-type embryos. (C) Degradation curves of Prpf31, AD5 and SP117 proteins (analyzed by Image J).

The human *PRPF31 *gene contains 14 exons and encodes a protein of 499 amino acids (aa). It has a predicted NOSIC domain found in RNA-binding proteins located at aa 93-144, a conserved RNA-binding NOP domain at aa 190-334 and a predicted nuclear localization signal (NLS) at aa 351-364 (Figure 1A, Additional file 1, Figure S1). In RP11 families, a multitude of mutations including missense and nonsense substitutions, deletions as well as insertions have been reported in the *PRPF31 *gene. For AD5, a 11 base pair (bp) deletion (1115-1125 del) in exon 11 results in a frameshift after amino acid (aa) 371 leading to a truncated protein of 469 residues (Additional file 1, Figure S1). In the case of the SP117 mutation, a 1 bp insertion (769-770 bp) leads to a frameshift after aa 256 and results in the addition of 21 missense residues (Additional file 1, Figure S1). Zebrafish (*Danio rerio*) Prpf31 (DrPrpf31) contains 508 amino acids and shares high similarity (82.0%) with human PRPF31. To generate zebrafish Prpf31 variants mimicking the human AD5 mutation, we used a truncated cDNA sequence encoding 382 aa retaining a predicted NLS (Figure 1A). In zebrafish *prpf31*, a 1 bp insertion at the same position as in human SP117 would also cause 21 missense residues which share 57.1% identity and 76.2% similarity to those in human SP117. Thus, corresponding to the human SP117 mutation, we inserted 1 bp between 801-802 bp in zebrafish *prpf31 *to generate a cDNA encoding a truncated protein with 21 missense residues after the predicted frameshift, which lacks the NLS present in AD5 and wild-type protein (Figure 1A).

### Distinct stabilities and activities of zebrafish AD5 and SP117 variants after mRNA injection *in vivo*

To analyze if mutant *prpf31 *mRNAs are translated into protein and how stable these mutant proteins are *in vivo*, we injected 61 nM mRNAs encoding wild-type Prpf31, AD5 and Sp117 into zebrafish embryos and examined protein levels with an anti-PRPF31 antibody using Western blot analysis (Figure 1B). A band at 56 kDa representing endogenous Prpf31 protein was detected, as well as both truncated proteins at their predicted sizes of 42 kD and 29 kD, respectively. We next compared the stability of wild-type Prpf31 to that of AD5 and SP117 variants by examining protein levels over a period of 29 hours post fertilization (hpf) (Figure 1B). After injection of identical doses of mRNAs into zebrafish embryos, we found that wild-type Prpf31 was still detectable at 24 hpf. In contrast, the SP117 protein was degraded quickly and was hardly detectable at 14 hpf. AD5 protein was degraded less rapidly than SP117 and could still be detected at 19 hpf.

Densitometric quantification of Western blots (Figure 1C) revealed degradation rates for Prpf31, AD5 and SP117. The different degradation rates of these proteins were not a consequence of differences in RNA stability as determined by qRT-PCR (Additional file 2, Figure S2). This suggested that the AD5 variant is more stable than SP117 and both of them are less stable than wild-type Prpf31.

**Figure 2:**
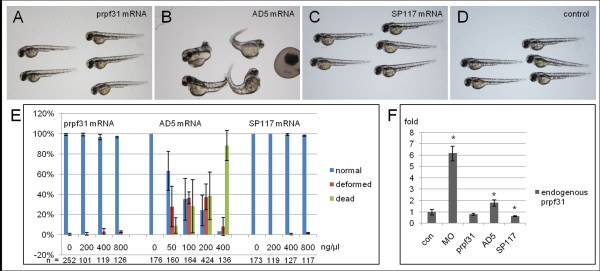
**Distinct activities of zebrafish Prpf31 AD5 and SP117 after mRNA injection**. (A) Wild-type phenotype in embryos injected with 800 ng/ul *prpf31 *mRNA. (B) Embryos injected with 200 ng/ul AD5 mRNA exhibit severe deformations (malformed brains, short trunks, cardiac edema, and curved body axis) or death. (C) Normal phenotype in embryos injected with 800 ng/ul SP117 mRNA. (D) Non-injected control embryos at 48 hpf. (E) Quantitative analysis of embryonic phenotypes (normal, deformed and dead) after injection of different doses of *prpf31*, AD5 and SP117 mRNAs. The mean percentage of embryos in each group was calculated from two or three independent experiments and standard deviation is shown by error bars. (F) Analysis of endogenous *prpf31 *RNA levels by qRT-PCR after injection of 61 nM *prpf31*, AD5, SP117 mRNAs and 5 μg/μl *prpf31 *Morpholino oligos (MO), respectively. Both MO and AD5 mRNA injected embryos show significant increase of endogenous *prpf31 *levels. Data were analyzed using T-test. Significant differences are indicated by asterisks.

To investigate whether the RP-linked Prpf31 mutants have any detrimental effects *in vivo*, overexpression experiments were performed by injection of *prpf31 *and the respective mutant mRNAs into 1-cell stage zebrafish embryos. The resulting phenotypes were assessed at 48 hpf. As a control, three different doses of wild-type *prpf31 *mRNA (200 ng/μl, 400 ng/μl, 800 ng/μl) were tested first. Almost all embryos showed normal phenotype even with the highest dose injected (Figure 2A,E), indicating that overexpression of wild-type *prpf31 *mRNA injection has no adverse effect on embryonic development in zebrafish.

When different doses of AD5 mRNAs were injected, the embryos showed increased embryonic lethality in a dose dependent manner (Figure 2E). 88.3% of embryos died after injection of 400 ng/μl mRNA. At lower doses (100 ng/μl), embryonic malformations were observed in 37.3% of embryos. The deformed embryos showed general morphological defects (Figure 2B), such as malformed brains, short trunks, cardiac edema, and curved body axis. This indicates that AD5 mRNA has adverse effects on embryonic development when overexpressed. In contrast, when different doses of SP117 mRNA were injected, embryos did not show any deformations (Figure 2C). Even at concentrations of 800 ng/μl, embryos developed normally (Figure 2E). This suggests that overexpressed SP117 variant has no effect on embryonic development.

We next tested whether endogenous p*rpf31 *expression was affected by injection of AD5 and SP117 mRNAs. By qRT-PCR, endogenous *prpf31 *expression was analyzed at 11 hpf after injection of 61 nM *prpf31*, AD5, SP117 mRNAs and 5 μg/μl *prpf31 *Morpholino oligos (MO), respectively. As shown in Figure 2F, embryos injected with the translation blocking *prpf31 *MO showed a significant increase in endogenous *prpf31 *transcripts, suggesting a possible compensatory mechanism to counteract reduced Prpf31 protein levels. Interestingly, embryos injected with AD5 mutant mRNA also resulted in an increase of endogenous *prpf31 *expression, albeit to a lesser extent. In contrast, embryos injected with *prpf31 *wild-type and SP117 mRNA showed no up-regulation regulation of endogenous *prpf31 *expression, but rather a down-regulation in case of SP117. Given that injection of both, antisense *prpf31 *MO as well as AD5 mRNA similarly resulted in an up-regulation of endogenous *prpf31 *transcription, possibly as a consequence of a mechanism to compensate for the loss of functional Prpf31 protein, we next tested the possibility that AD5 acts in a dominant-negative fashion by using rescue experiments in zebrafish embryos.

### Rescue of *prpf31 *morphant phenotypes by wild-type but not mutant *prpf31 *mRNA

**Figure 3:**
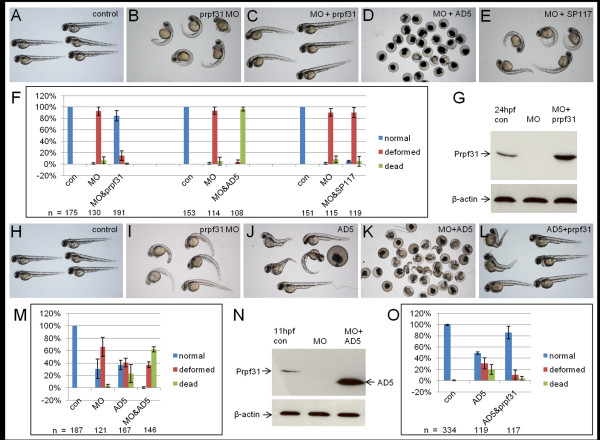
**Rescue of *prpf31 *morphant and AD5 phenotype**. (A-G) Rescue of *prpf31 *morphant phenotype by wild-type *prpf31*, AD5 and SP117. (A) Non-injected control larvae at 48 hpf. (B) Morphants injected with 10 μg/μl (1.2 mM) *prpf31 *MO show severe malformations, such as reduced brain and eye size, cardiac edema and curved tails. (C) Full rescue of morphant phenotype after co-injection of 573 ng/μl (0.93 μM) *prpf31 *mRNA. (D) Embryos co-injected with 437 ng/μl (0.89 μM) AD mRNA show high lethality. (E) Embryos co-injected with 325 ng/μl (0.84 μM) SP117 mRNA showed deformations similar to the situation in morphants. (F) Quantitative analysis of rescue efficiency with different MO/RNA combinations. The mean percentage of embryos in each group was calculated from three independent experiments and standard deviation is shown by error bars. (G) Western blot shows reduced endogenous Prpf31 levels after MO injection. Co-injection of *prpf31 *mRNA results in elevated Prpf31 levels at 24 hpf. (H-O) Co-injection of AD5 with low doses of *prpf31 *MO and *prpf31 *RNA. (H) Control embryos at 48 hpf. (I) Morphants injected with 5 μg/μl *prpf31 *MO. (J) Embryos injected with 200 ng/μl AD5 mRNA. (K) Embryos co-injected with *prpf31 *MO and AD5 mRNA. (L) Embryos co-injected with 200 ng/μl AD5 mRNA and 262 ng/μl *prpf31 *mRNA. (M) Quantitative analysis of embryos injected as in H-K. Co-injection of MO and AD5 mRNA results in a significant increase in lethality. (N) Western blot showing almost undetectable endogenous Prpf31 levels after co-injection of 200 ng/μl AD5 mRNA and 5 μg/μl *prpf31 *MO, but abundant AD5 protein at 11 hpf. (O) Quantitative analysis of embryos injected with 200 ng/μl AD5 and 262 ng/μl *prpf31 *mRNA. Co-injection of *prpf31 *mRNA resulted in a partial rescue of the AD5 induced phenotype.

To investigate whether the truncated Prpf31 variants lose or retain their *in vivo *function or possibly act in a dominant-negative fashion, rescue experiments were performed. Endogenous Prpf31 expression was reduced by the injection of translation blocking MOs into zebrafish embryos as described before [15] and all resulting phenotypes were assessed at 48 hpf. As reported earlier, low doses of *prpf31 *MO result in a deficient optokinetic nystagmus (OKN) response, whereas high MO doses lead to broad and severe malformations and increased embryonic lethality as Prpf31 is a generally required splice factor [15]. In the present study, we used high doses of Prpf31 MO for co-injection with various RNAs as this allowed an efficient assessment of embryonic rescue based on gross morphology. Consistent with previous reports [15], knock-down of *prpf31 *after injection of high MO concentrations (10 μg/μl) resulted in severe malformations in the injected embryos (Figure 3B,F). The morphants showed reduced brain and eye size, cardiac edema and curved tails at 48 hpf. The majority died after 6 dpf, indicating that Prpf31 function is essential for embryonic survival. Co-injection of *prpf31 *mRNA at 573 ng/μl with silent mutations in the MO binding site resulted in a rescue of malformations and reversion to wild-type phenotype in 84.6% of injected embryos (Figure 3C,F; see also [15]). Embryos with normal eye size and extended trunks and tails were scored as rescued embryos (embryos on the right in Figure 3C), while embryos with small eyes and curved tails were scored as not rescued (embryos on the left in Figure 3C). Prpf31 protein was almost undetectable by Western blot analysis in MO injected embryos, while in embryos co-injected with *prpf31 *mRNA elevated Prpf31 levels were present (Figure 3G). Importantly, co-injection of AD5 mutant mRNA at similar doses had no rescue effect and instead resulted in early embryonic lethality in all embryos injected (Figure 3D,F). In contrast, co-injection of SP117 mutant mRNA together with MO showed no significant difference to embryos injected with MO alone (Figure 3E,F). Thus, both AD5 and SP117 mutant mRNAs are not able to rescue the *prpf31 *morphant phenotype and instead AD5 mutant RNA aggravates the MO phenotypes.

To further investigate whether AD5 has a dominant-negative effect, we injected lower doses of *prpf31 *MO (5 ng/μl) together with AD5 mRNA (200 ng/μl) into zebrafish embryos. This resulted in a significant increase in embryonic lethality (62.2%), when compared to AD5 mRNA injection alone (22.7%; Figure 3J,K,M). Western blot analysis showed that endogenous Prpf31 protein levels were reduced in morphants and exogenous AD5 was expressed at high levels in co-injected embryos (Figure 3N). This provided further support that AD5 has dominant-negative activity. As AD5 may have the ability to compete with endogenous Prpf31 protein, we speculated that increased levels of wild-type Prpf31 may rescue AD5 induced defects. We therefore injected wild-type *prpf31 *mRNA together with AD5 mRNA and analyzed for possible rescue of AD5 induced lethality and deformations. Injection of 200 ng/μl (0.4 μM) AD5 mRNA resulted in typical deformations in 31% of injected embryos and a lethality rate of 20%. Co-injection of 262 ng/μl (0.4 μM) *prpf31 *mRNA resulted in a partial rescue of embryonic defects (wild-type phenotype in 85.4% embryos) and a reduction of lethality to 4.0% (Figure 3L,O). As shown in Figure 3L, rescued embryos showed extended trunks and tails (embryos on the right), while embryos with curved tails were scored as not rescued (left) as before. This indicates that increasing amounts of wild-type *prpf31 *mRNA can rescue AD5 induced embryonic lethality.

### Subcellular localization of wild-type and mutant Prpf31 variants in rod photoreceptors

**Figure 4:**
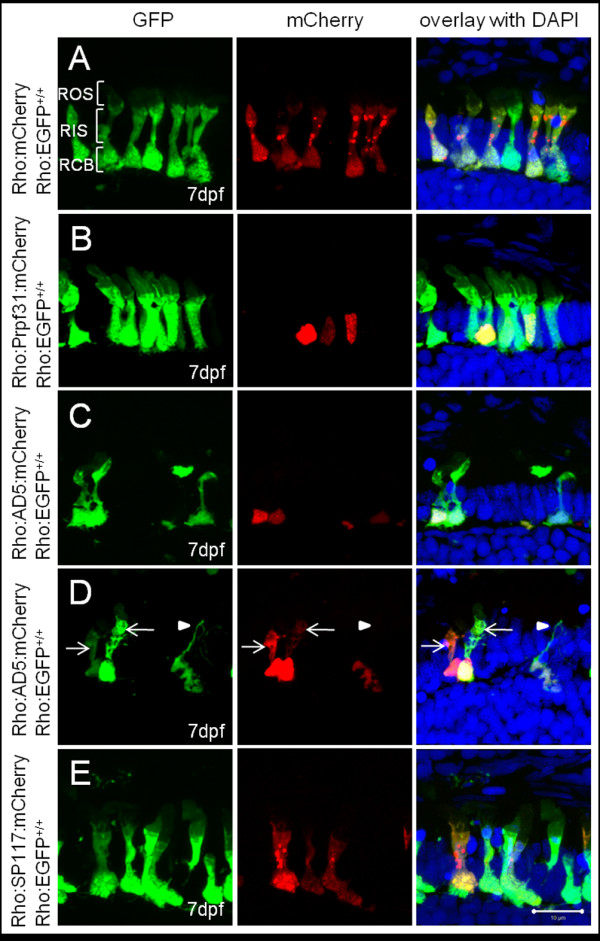
**Subcellular localization of wild-type and mutant Prpf31 fusion proteins in rod photoreceptors**. Wild-type and mutant Prpf31 fused to mCherry and driven by the rhodopsin promoter were transiently expressed in 7 dpf Tg(Rho:EGFP) larvae in which EGFP is stably expressed in rods and distributed throughout the cytoplasm. (A) Control mCherry was localized in the rod cytoplasm similar as EGFP. (B) Wild-type Prpf31:mCherry was localized in the nuclei of rods. (C) AD5:mCherry was mainly detected in the nuclei of rods similar to wild-type Prpf31. (D) In exceptional cases, AD5:mCherry was also observed in the cytoplasm of individual rods (arrows). Some of the AD5:mCherry positive rods show loss of the outer segment and disintegrated nuclei (arrow head) indicative for rod degeneration. (E) SP117:mCherry was aberrantly localized in the cytoplasm of rods rather than the nucleus. Morphology, however, is normal in SP117 expressing rods. ROS, rod out segment; RIS, rod inner segment; RCB, rod cell body. Left column shows EGFP channel, middle column shows mCherry channel and right column shows overlay together with DAPI stain.

To determine Prpf31 protein localization and function *in vivo*, we transiently expressed fusion proteins encompassing wild-type or mutant Prpf31 variants fused in frame to mCherry under the control of the rhodopsin promoter in rod photoreceptors of zebrafish. mCherry driven by the same rhodopsin promoter was used as control. These constructs were injected into Tg(Rho:EGFP) embryos, in which EGFP is expressed exclusively in rod photoreceptors. In Tg(Rho:EGFP) retinas (Figure 4, green channel), rod cell bodies, inner as well as outer segments could be clearly distinguished on cryosections at 7 dpf. As shown in green channel of Figure 4A, EGFP was distributed throughout the cytoplasm, with high levels in the cell body and inner segment and slightly fainter expression in the outer segments. In the red channel shown in Figure 4A, the Rho:mCherry control was found in the rod cytoplasm. Spots of accumulated mCherry were observed in inner segments. In Figure 4B, embryos transiently expressing Rho:Prpf31:mCherry showed Prpf31:mCherry protein localized to the nuclei. This is consistent with other reports on subcellular localization of Prpf31 in the nucleus [29-32]. Importantly, AD5:mCherry was also localized in the nuclei of rod photoreceptors similar to wild-type Prpf31 (Figure 4C). Only in few exceptional cases (Figure 4D, arrow), AD5:mCherry protein was observed in the cytoplasm (rod inner segment) indicating aberrant subcellular localization of the mutant protein. Such rods had intact inner and outer segments. Some AD5:mCherry positive rods, however, showed loss of the outer segment and disintegrated nuclei (Figure 4D, arrow head) possibly indicating that AD5 expression may lead to rod degeneration. Transient expression of SP117:mCherry resulted in aberrant localization of this mutant variant to the cytoplasm rather than the nucleus in all examined cases (Figure 4E). No obvious change in the general morphology was observed in SP117:mCherry expressing rods.

### Rod photoreceptor degeneration in transgenic fish stably expressing AD5

**Figure 5:**
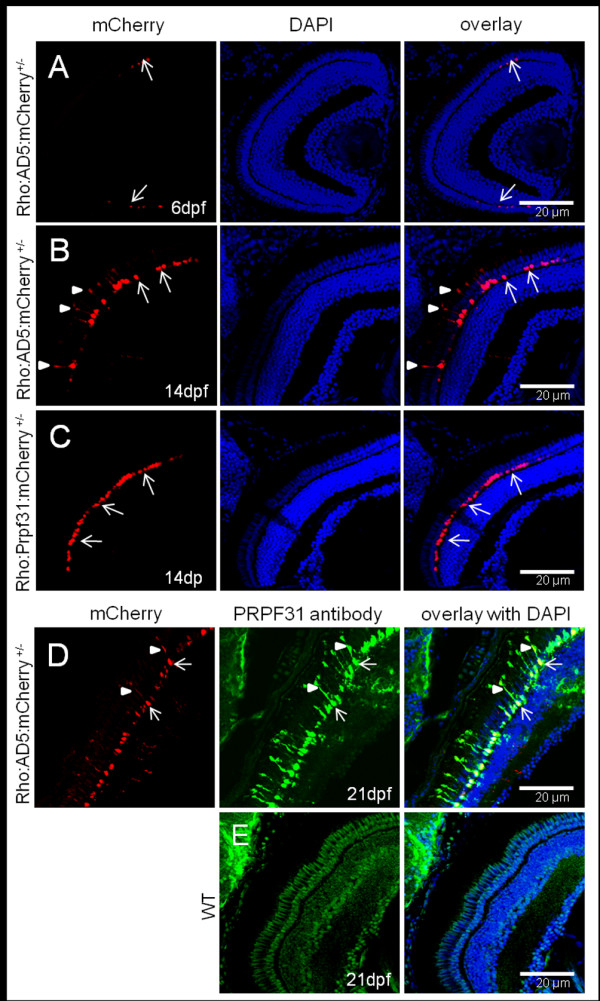
**Accumulation of AD5 in rod cytoplasm analyzed in Tg(Rho:AD5:mCherry) larvae at different stages**. (A) At 6 dpf, AD5:mCherry was exclusively localized to the nuclei of rods (arrows). (B) In contrast, AD5:mCherry was found also in the inner segments (arrowheads) of rods at 14 dpf. (C) Tg(Rho:Prpf31:mCherry) transgenic larvae at 14 dpf were used as control to show that Prpf31:mCherry is only found in rod nuclei (arrow) but not the cytoplasm. (D) In 21 dpf Tg(Rho:AD5:mCherry) larvae, AD5 protein as detected by PRPF31 antibody (green) was co-localized with mCherry (red) in both the nucleus (arrow) and rod inner segment (arrowhead). (E) In 21 dpf wild-type fish, endogenous Prpf31 protein was localized in all nuclei. Note that imaging exposure in E was extended compared to D to reveal nuclear localization of Prpf31 in all retinal cells. Shorter exposure in D indicates strong expression of transgenic AD5 under control of the rhodopsin promoter in rod photoreceptors.

To investigate further how the AD5 mutation affects rod photoreceptor cells, we generated stable transgenic fish expressing AD5:mCherry or Prpf31:mCherry fusion proteins exclusively in rods under control of the rhodopsin promoter. At 6 dpf, AD5:mCherry was entirely localized to the nuclei of rods (Figure 5A). At 14 dpf, however, AD5 was found not only in nuclei but also in the inner segments of rods (Figure 5B). This recapitulates the cytoplasmic localization of AD5 that we observed previously in few larvae transiently expressing AD5 (Figure 4D). As a control, in 14 dpf Tg(Rho:Prpf31:mCherry) transgenic larva, Prpf31 was only found in nuclei but not the cytoplasm indicating correct localization of the fusion protein (Figure 5C).

To investigate if re-distribution of AD5 to the cytoplasm results from partial proteolysis which may digest the AD5:mCherry fusion protein and release mCherry to the cytoplasm, we determined AD5 protein distribution using immunostaining with the Prpf31 antibody. As shown in Figure 5D, AD5 protein as detected by Prpf31 antibody staining was co-localized with mCherry in both the nucleus and rod inner segment, while in wild-type control endogenous Prpf31 protein was only localized in nucleus (Figure 5E). This indicates that AD5:mCherry was mislocalized in the cytoplasm as intact fusion protein.

**Figure 6:**
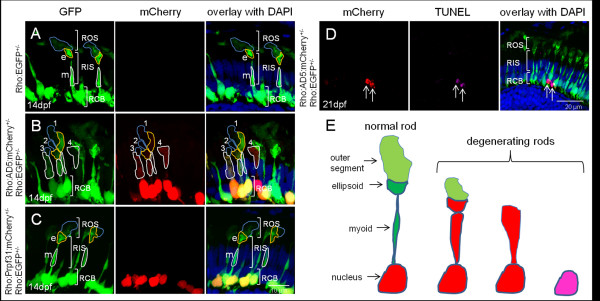
**Different degrees of rod photoreceptor degeneration visualized in stable transgenic fish expressing AD5**. (A) In control heterozygous Tg(Rho:EGFP)^+/- ^larva (F1) at 14 dpf, abundant EGFP is detected in rod cell bodies and inner segments including the proximal myoid region (white border, m) and distal ellipsoid region (yellow border, e), both of which show distinct morphology. The outer segment (blue border) has faint EGFP. Abbreviations are the same as used in Figure 4. (B). In double transgenic fish, expressing AD5:mCherry, rods show morphological degeneration to different extents at 14 dpf and AD5:mCherry is found to be distributed in the inner segments of many rods. (C) In contrast, double transgenic larvae expressing Tg(Rho:Prpf31:mCherry) show normal rod morphology at 14 dpf. (D) TUNEL assay to detect apoptotic cells in double transgenic fish at 21 dpf expressing AD5:mCherry. Rods undergoing apoptosis lack both outer and inner segments. (E) Model showing different rod degeneration phenotypes observed after AD5 expression. Initially, AD5:mCherry (red) is found in rod nuclei. Later, AD5 can also be found in rod inner segments. Affected rods lose their outer segments, and myoid and ellipsoid fuse. Several rods were observed to enter the final phase of apoptosis when DNA is fragmented and can be detected by TUNEL staining.

We next crossed the Tg(Rho:EGFP) line with either Tg(Rho:AD5:mCherry) or Tg(Rho:Prpf31:mCherry) as control to generate double transgenic lines. In the Tg(Rho:EGFP) control fish at 14 dpf, distinct rod morphology was observed (Figure 6A). EGFP was strongly expressed in rod cell bodies. The inner segments were elongated and the proximal myoid region as well as the distal ellipsoid region both contained abundant EGFP protein. The outer segment had fainter EGFP signal than the ellipsoid region of the inner segment. In double transgenic fish expressing AD5:mCherry, the rods showed different extents of morphological degeneration, as shown exemplary for one specimen in Figure 6B. Several rods showed AD5:mCherry localization in cytoplasm (rods 1-4 in Figure 6B). Some rods had normal shape with intact outer segments, ellipsoid, myoid and cell bodies (rod 1). Others had reduced outer segments, while ellipsoid and myoid were still intact (rod 2). There were also rods that showed reduced outer segments, and ellipsoid and myoid regions appeared fused (rod 3). Finally, other rods lost their outer segments and ellipsoid and myoid regions were fused and became indistinguishable (rod 4). Importantly, in controls at 14 dpf when Prpf31:mCherry was expressed in a stable transgenic setting, all rods showed normal morphology (Figure 6C).

One possibility to explain why AD5 relocated to the cytoplasm of rods is that AD5 expression causes apoptosis of rods which leads to nuclear membrane disintegration and release of AD5:mCherry into the cytoplasm. To test this possibility, we performed TUNEL assays in transgenic fish at 21 dpf stably expressing AD5. Several TUNEL positive cells undergoing apoptosis and expressing AD5 were detected (Figure 6D). We observed that these rods already lost their outer and inner segments, which is consistent with the fact that TUNEL assay detects fragmented DNA during late phases of the apoptosis process. This also suggests that relocation into the cytoplasm represents an early stage of rod degeneration. To determine the extent of apoptosis, we analyzed three cryo-sections each containing two retinas from transgenic fish at 21 dpf expressing Rho:AD5:mCherry and Rho:EGFP, Rho:Prpf31:mCherry and Rho:EGFP as well as Rho:EGFP fish as control (Additional file 3, Figure S3). We counted a total of 29 apoptotic cells on three sections obtained from Rho:AD5:mCherry and Rho:EGFP double-transgenic fish, while 5 and 4 apoptotic cells were detected in control fish expressing Prpf31 and GFP, respectively. This suggests that expression of AD5 results in an increase of cell death in photoreceptor cells. AD5 expressing rods appeared dysmorphic and their appearance may reflect morphological changes that are correlated with increased cell death (Figure 6E).

**Figure 7:**
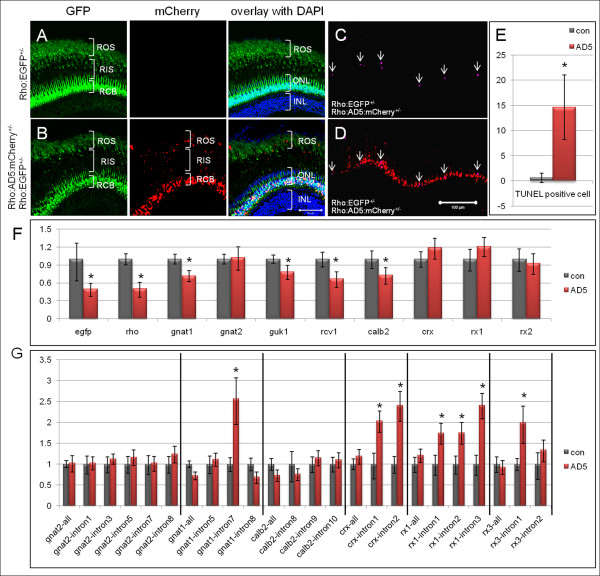
**Rod degeneration and splicing defects detected in adult AD5 transgenic fish**. (A-E) Rod degeneration in adult AD5 transgenic fish. (A) Adult retina of Tg(Rho:EGFP) control fish at 5 months. EGFP is found in cell bodies (RCB) and outer segments (ROS), and can be seen faintly in inner segments (RIS). (B) Double transgenic fish expressing AD5:mCherry show multiple mCherry positive dots in the rod inner and outer segments, in addition to nuclear mCherry. (C) TUNEL positive apoptotic cells (arrow) in double transgenic fish expressing AD5:mCherry. (D) Overlay of TUNEL signal in image C with AD5:mCherry signal. (E) Number of TUNEL positive cells in double transgenic fish at 5 months expressing AD5:mCherry and Tg(Rho:EGFP) and in control fish (Tg(Rho:EGFP)). (F) qRT-PCR analysis showing gene expression profiles in double transgenic fish retinas expressing AD5:mCherry. 5 months old Tg(Rho:EGFP) transgenic fish retinas were used as control. Transcript numbers of several genes involved in photo-transduction are reduced, including *rho*, *gnat1*, *guk1*, *recoverin*, and *calb2*. egfp expression is also reduced, consistent with our histological findings. The cone specific *transducin *(*gnat2*) and several retina specific transcription factors (*crx*, *rx1 *and *rx3*) show no significant change. (G) Detection of splicing defects in double transgenic fish retinas (Tg(Rho:EGFP × Rho:AD5:mCherry)) by qRT-PCR. Among the six analyzed transcripts, introns from four genes showed significant stabilization compared to the same introns in control fish (Tg(Rho:EGFP), which includes intron 7 of *gnat1*, intron 1 and intron 2 of *crx*, intron 1, intron 2 and intron 3 of *rx1*, and intron 1 of *rx3*. In contrast, intron levels for cone specific gene *gnat2 *showed no significant change when compared to the control. Data were analyzed by T-test. Significant differences are indicated by asterisks. ONL, outer nuclear layer; INL, inner nuclear layer.

In adult Tg(Rho:EGFP) control fish at 5 months (Figure 7A), abundant EGFP was detected in cell bodies and outer segments (ROS), while the inner segment (RIS) is too thin to observe faint EGFP expression. In the retinas of double transgenic fish expressing AD5:mCherry in addition (Figure 7B), multiple mCherry positive dots were detected in rod inner and outer segments at the dorsal periphery, possibly indicating areas where degeneration is in process. In these double transgenic fish, retina lacked the compacted organization of rod outer segments that is usually observed in control fish (Figure 7A), indicating possible degeneration of several outer rod segments. Consistent with this, multiple TUNEL positive apoptotic cells were detected in these double transgenic fish (Figure 7C, D). Cell counts revealed that the number of apoptotic cells was significantly increased in AD5 expressing fish when compared to controls (Figure 7E). Although the rod outer segment layer was severely affected in AD5 expressing transgenic fish, the rate of apoptotic rod cells was surprisingly low, with about 15 apoptotic cells per 400 mCherry positive cells. This opens the possibility that the majority of rods in the AD5 expressing retina are dysfunctional with outer segment undergoing degeneration and that cell death is a long-term process in these specimens.

### Reduced expression of retina transcripts and splicing defects in AD5 transgenic retinas

Splice factor deficiencies lead to alterations in the transcriptome of affected retinas [15] that could be attributed to aberrant splicing. To detect changes in gene expression profiles indicative for rod photoreceptor degeneration, qRT-PCR analysis of selected retina genes was performed in adult retinas dissected from double-transgenic fish. Tg(Rho:EGFP) transgenic fish were used as control. Expression of many genes involved in phototransduction appeared down-regulated in AD5 expressing retinas, including rho, gnat1, guk1, rcv1, calb2 (Figure 7F), but we can not exclude that a reduction in rod numbers is responsible for this effect. Using qRT-PCR, we also observed reduced *egfp *expression, which is consistent with our histological findings described above. Interestingly, *gnat2*, the cone specific *transducin *and several retina specific transcription factors including *crx*, *rx1 *and *rx3*, showed no significant change, suggesting that only a subset but not all retina transcripts are affected by AD5 expression.

Finally, we examined whether mRNA splicing is affected in AD5 expressing retinas using intron-specific qRT-PCR for six selected genes (Figure 7G). Among the analyzed transcripts, introns from four genes showed significant stabilization compared to control, which includes intron 7 of *gnat1*, intron 1 and intron2 of *crx*, intron 1, intron 2 and intron 3 of *rx1*, and intron 1 of *rx3*. In contrast, intron levels for cone specific gene *gnat2 *showed no significant change when compared to the control. Given that several transcripts were affected in our small-scale assay, this opens the possibility that the AD5 mutant variant selectively affects splicing of a subset of transcripts in rod possibly by interfering with wild-type Prpf31 in a dominant-negative fashion.

## Discussion

Mutations in the ubiquitously expressed splicing factor PRPF31 are found in 8% of patients suffering from autosomal-dominant Retinitis Pigmentosa (ad-RP; [1]). In our study, we used the zebrafish model to investigate the etiology of two RP mutations in the *PRPF31 *gene, AD5 and SP117. While both mutations cause the same disease with identical symptoms, our data suggest that this is caused by two fundamentally different mechanisms. We show that transgenic expression of SP117 leads to aberrant localization of the mutant protein in the rod cytoplasm rather than nucleus, but does not affect rod morphology or numbers. This implicates a loss-of-function for the SP117 mutation which results in reduced U4/U6.U5 tri-snRNP assembly and suggests that in patients carrying this mutation the disease phenotype is caused by haploinsufficiency. On the other hand, our studies using over-expression of AD5 strongly suggest that this variant has dominant-negative activity and its expression results in the degeneration of rod outer segments and late onset apoptosis (see below).

This study represents the first *in vivo *analysis to show how *PRPF31 *mutations lead to photoreceptor degeneration. We report aberrant splicing in pre-mRNA transcripts using an animal model for RP that is easily accessible for bioimaging, high throughput genetic approaches and drug screening.

### Splicing factor deficiencies lead to RP

PRPF31 and at least four other RP related PRPF factors (PRPF3, PRPF8, PAP1, SNRN200) play essential roles in pre-mRNA splicing. Three hypothetical mechanisms are presently discussed to explain how mutations in general splicing factors can lead to photoreceptor specific degeneration. First, null mutations result in hyploinsufficiency by loss of function of the mutant protein or degradation of mutant mRNA by nonsense-mediated mRNA decay (NMD) [34-36]. As a consequence, the splicing machinery becomes compromised in its efficiency and hence cannot fulfil all its functions. Second, mutations lead to proteins with dominant-negative activities. These proteins may interfere with splicing and also potential other cellular activities, thereby leading to damage of the affected tissue. Third, mutations might lead to proteins forming insoluble and cytotoxic aggregates. Proteins affected by this class of mutations may affect the tissue by a combination of loss-of-function and dominant-negative effects. Consistent with the third mechanism, a missense mutation in *PRPF31*, A216P, has been reported to lead to insoluble protein that is deposited in the cytoplasm [30].

### SP117 and AD5 mutations cause photoreceptor degeneration by different modes

PRPF31 is ubiquitously expressed and sufficient levels are crucial for organ maintenance. Consistently, homozygous *Prpf31 *knock-out mice are embryonic lethal [33], whereas heterozygous *Prpf31 *knock-out mice surprisingly display degeneration of the retinal pigment epithelium (RPE) [8]. Thus, different PRPF31 thresholds either lead to degeneration or lethality. Reduced PRPF31 protein levels were detected in lymphoblastoid cells of RP patients, suggesting that haploinsufficiency is one of the possible causes for RP [11,37].

The SP117 mutation in *PRPF31 *results in premature termination before the NLS and has been reported to confer gain-of-function toxicity in cultured mouse retina cells, leading to reduced rhodopsin expression and apoptosis [31,32]. In our *in vivo *study, SP117 was found distributed along the rod cytoplasm rather than the nucleus, which is consistent with reports on mouse retina cell culture experiments. However, we found that expression of SP117 in zebrafish rods does not have detrimental effects on rod morphology or survival. Even over-expression at high doses in early embryos did not result in increased lethality. Instead, we found that SP117 protein is significantly less stable than wild-type or AD5 mutant Prpf31. This indicates that other than proposed from cell culture studies, mutant SP117 may not show any toxicity. We instead suggest that SP117 protein is non-functional, unstable and mislocalized, consequently leading to reduced tri-snRNP levels and hence a weakening of the spliceosome in a mutant situation. Thus, haploinsufficiency appears to be responsible for photoreceptor cell degeneration in human RP patients carrying the SP117 mutation.

Recently, it was reported that RP mutations in *PRPF3 *and *PRPF8 *exert dominant-negative activity in knock-in mice (*Prpf3*^+/T494M ^and *Prpf8*^+/H2309P^) and induce degenerative processes that however surprisingly are found in the retinal pigment epithelium and not photoreceptors [8]. With our study, we provide evidence that also AD5 acts in a dominant-negative fashion. The AD5 mutation has been intensively studied in several cell culture models. Using lymphoblastoid cells from RP patients, Rio Frio et al. could not detect any truncated AD5 protein by Western analysis and therefore concluded that AD5 leads to RP by haploinsufficiency upon nonsense-mediated decay of the mutant RNA [29]. However, this study did not analyze retina tissue and the expression pattern in lymphoblastoid cells may not represent the situation in photoreceptor cells where symptoms manifest. On the other hand, in studies using cultured mouse retinal cells, AD5 expression has been reported to result in reduced rhodopsin expression, increased apoptosis and splicing defects in artificial minigenes [31,32] very similar to the findings in our *in vivo *model.

We provide four lines of evidence supporting that AD5 exhibits dominant-negative effect. First, AD5 over-expression leads to early embryonic lethality in a dose-dependent fashion in zebrafish embryos. This lethality can be partially rescued by co-expression of wild-type *prpf31*. Second, AD5 over-expression aggravates the effect of Morpholino-induced *Prpf31 *knock-down. Third, AD5 expression in zebrafish embryos shows the same effect on endogenous *prpf31 *transcript levels, as well as other retina-specific transcripts as the Morpholino induced gene knock-down of *prpf31 *[15]. Finally, rod-specific AD5 expression in stable transgenic fish results in the degeneration of rod outer segments and late onset apoptosis. Thus, we propose that mutant AD5 competes with endogenous PRPF31 for binding in the U4/U6.U5 tri-snRNP eventually leading to an impairment of the tri-snRNP and decreased splicing activity. Consequently, RP in patients carrying only one functional *PRPF31 *allele might be caused through a combination of dominant-negative effects and haploinsufficiency.

### Different degrees of rod photoreceptor degeneration visualized in AD5 transgenic zebrafish

*Prpf31 *knock-out and A216P knock-in mouse have been generated, however, none of them showed degeneration in the photoreceptor layer, but instead in the retinal pigment epithelium [8,33]. In contrast, our AD5 stable transgenic fish model revealed different degrees of rod degeneration as well as photoreceptor death (Figure 6E) very similar to the symptoms observed in human RP patients. Thus, our data provide an *in vivo *model in which the AD5 mutation of Prp31 leads to degeneration in photoreceptor cells. We found that early AD5 protein was restricted to rod nuclei. At later stages, however, AD5 was often found in the inner segments possibly as a consequence of nuclear membrane disintegration or alternatively deficient nuclear import. Dysfunctional rods with degenerated outer segments were observed as well as cells expressing apoptotic markers and lacking both outer and inner segments. The relatively low percentage (3.7%) of identified apoptotic rods in adult AD5 transgenic fish suggests that the induced rod degeneration is a long-term process, possibly reflecting the typical features of adult onset in RP patients. It is possible that compensatory mechanisms, e.g. the up-regulation of endogenous prpf31 and rod regeneration, account for the observed low percentage of apoptosis. Fish continuously generate photoreceptor cells throughout their life and it has been reported that chronic rod cell death stimulates rod genesis [38]. This may suggest that an increased number of newly generated rods in AD5 transgenic fish could mask the actual extent of rod cell death.

### Splicing defects in AD5 expressing zebrafish retinas

We have previously shown that a *prpf31 *knock-down in zebrafish leads to the down-regulation of retina transcripts, many of which are implicated in RP pathogenesis or components of the photoreceptor specific transcription factor network [15]. Here, we report for the first time splicing defects in some of these retina-specific transcripts caused by transgenic expression of AD5 *in vivo*. Screening a small panel of photoreceptor specific genes, we identified splicing defects in *gnat1*, *crx*, *rx1 *and *rx3*. *gnat1 *encodes the rod specific form of transducin (Trα), an essential component in the rod phototransduction cascade [39-41]. For *Gnat1*, loss or deterioration of rod function was reported in homozygous knock-out mice [39] and in transgenic mice expressing mutant TrαG38D [40] and TrαQ200 L [41]. *crx *encodes a cone-rod homeobox protein, a retina specific transcription factor important for maintaining photoreceptor function [42]. *Crx *null mice failed to form outer segments of photoreceptor cells resulting in photoreceptor degeneration [43]. Most notably, *Crx *is a known RP causing gene and Crx mutations have been identified in RP patients [7]. Finally, retinal homeobox (*Rx/Rax*) genes, *rx1 *and *rx3*, are essential for eye development [44,45]. Loss of Rx function prevents eye formation (anophthalmia) in fish and mice [46-48]. In humans, mutations in *RX *lead to anophthalmia or microphthalmia [49]. Hence, all genes with splicing defects identified in this study are crucial for the formation and/or maintenance of photoreceptor function. This opens the possibility that aberrantly spliced *gnat1*, *crx*, *rx1 *and *rx2 *contribute to the rod degeneration observed in AD5 transgenic fish.

In addition to splicing defects, we also observed reduced transcription of several other photoreceptor-specific genes very similar to the situation observed in zebrafish *prpf31 *morphants [15]. We suggest that it is the combination of wrongly spliced as well as diminished transcripts that eventually causes rod degeneration. For the future, our model is also excellently suited to study the effect of degenerative processes in rods on neighboring cells. This is important as in RP patients cones are secondarily affected in a process known as bystander-associated cell death [50].

### Hypothesis for the retina-specific phenotype

The retina is a fast-metabolizing tissue which has a high demand for correctly spliced transcripts. Reduced levels of functional tri-snRNPs due to mutations in PRPF31 may still be sufficient for most of the general cell types, but not adequate for highly demanding photoreceptor cells. This could lead to a situation where reduced splicing efficiency leads to the accumulation of defects mostly in the retina, eventually resulting in a retina-specific phenotype while other tissues are not affected. Consistent with this, we recently showed that only low levels of Prpf31 are required for maintenance of general organ development while retina development requires more Prpf31 [15]. Alternatively, individual mRNAs that are problematic to splice under such conditions could also be the primary cause for retina-specific defects [15]. In the present study, we detected splicing defects in retinas expressing mutant *Prpf31*. Our observation that only single introns of individual retina transcripts are affected by aberrant splicing suggests that a partial Prpf31 deficiency might indeed selectively affect splicing of a distinct subset of transcripts. The fact that one of these transcripts encodes Rx1, a retina-specific transcription factor, implicates that this could affect a transcriptional network consequently leading to a photoreceptor specific phenotype. Noteworthy, our *in vivo *approach has limitations as it used rod specific expression of a Prpf31 mutant (for comparison of expression levels of mutant versus endogenous Prpf31, see Additional file 4, Figure S4). Therefore, we cannot exclude that there are splicing defects also in other tissues under conditions of general Prpf31 deficiency that obviously, however, do not interfere with the function of the respective organ. Definitely, more work is required in the future to determine the mechanisms that underlie this interesting phenomenon. In addition, as the very strong rhodopsin promoter is used to drive expression of the SP117 and AD5 RNAs, there is a possibility that the levels of mutant proteins, AD5 in particular, may contribute to the changes observed.

## Conclusions

At present, no therapy is available to efficiently treat RP in patients. Our results show that different *PRPF31 *mutations act through distinct mechanisms, i.e. loss-of-function versus dominant-negative activity. We show that providing wild-type Prpf31 is able to rescue the detrimental effect caused by AD5 expression in zebrafish embryos. This opens the possibility that any elevation of the wild-type *PRPF31 *allele may prevent or delay degeneration of photoreceptor cells. Future studies on the transcriptional or post-transcriptional control of *PRPF31 *could hence lead to new avenues towards novel RP therapies.

## Materials and methods

### Plasmid construction and Morpholinos

Full-length zebrafish *prpf31 *was amplified from cDNA generated from 3 dpf embryos using a First Strand Reverse Transcriptase Kit (Fermentas). The zebrafish AD5 mutant was generated by PCR amplification of a fragment comprising the N-terminal 382 aa residues. The SP117 mutant was generated by site-specific PCR mutagenesis as described previously [51] except that three rounds of PCR were used (for details see Additional file 1, Figure S1). Capped mRNAs were transcribed *in vitro *by using the mMessage mMachine kit (Ambion), and purified using RNeasy Mini columns (Qiagen).

Translation blocking antisense Morpholino oligos (MOs) directed against the start ATG of zebrafish *prpf31 *were designed and synthesized by Gene-Tools. To prepare mRNAs for rescue experiments, with six silent mutations around the ATG were used for amplification of *prpf31*, AD5 and SP117. DNA fragments were subcloned into pCS2+ for capped mRNA synthesis.

Five plasmids were used for transgene generation. Rho:EGFP and Rho:mCherry plasmids were constructed using a 1.2-kb upstream promoter region of zebrafish rhodopsin [52-54] and full length EGFP or mCherry coding sequences. Fragments were subcloned into the pBluescript II (SK+; Stratagene) flanked by I-SceI sites. Then Prpf31, AD5 and SP117 coding sequences lacking their stop codons were subcloned in-frame into Rho:mCherry to generate Rho:Prpf31:mCherry, Rho:AD5:mCherry and Rho:SP117:mCherry constructs, respectively. Sequences of all primers are listed in Additional file 5, Table S1.

### RNA injections

Several injection strategies were used. To detect stability of Prpf31 and its mutant variants, 61 nM *prpf31*, AD5 and SP117 mRNAs were injected into zebrafish embryos at the 1 to 2-cell stage. To detect changes of endogenous *prpf31 *expression, 5 mg/ml *prpf31 *MO and 61 nM *prpf31*, AD5 and SP117 mRNAs were injected into embryos. To examine toxicity of Prpf31 variants, 200, 400, 800 ng/μl *prpf31 *and SP117 mRNA, as well as 50, 100, 200, and 400 ng/μl AD5 mRNA were injected. For rescue experiments, 830-930 nM mRNAs of *prpf31 *and its mutants (*Prpf*31 mRNA: 573 ng/μl; AD5 mRNA: 473 ng/μl; SP117 mRNA: 325 ng/μl) with silent mutations at ATG regions were co-injected with 10 μg/μl *prpf31 *MO. To test dominant-negative activities of AD5, 200 ng/μl AD5 mRNA with silent mutations at ATG regions was co-injected with 5 μg/μl *prpf31 *MO. To rescue AD5 phenotype, 262 ng/μl *prpf31 *was co-injected with 200 ng/μl AD5 mRNA. Between 0.5 to 1 nl of solutions were injected into zebrafish embryos at the one-cell stage in all experiments described.

### Generation of transient and stable transgenic fish

Plasmids Rho:EGFP, Rho:Prpf31:mCherry, Rho:AD5:mCherry, Rho:SP117:mCherry, and Rho:mCherry were purified by Qiagen midi-prep kit (Qiagen) and used for transient expression or transgene generation. An injection solution was prepared following the I-SceI protocol [55]: purified plasmid DNA at 60 ng/μl (final concentration); I-SceI meganuclease (NEB), 7.5 U; 10× I-SceI buffer, 3 μl; water to final volume of 30 μl. The solution was incubated at room temperature for 1 hour before injection. Injected embryos were screened for EGFP or mCherry fluorescence in the eyes after 4 dpf. Embryos for stable line generation were raised to sexual maturity and crossed with wild-type fish for screening. In this study, three stable transgenic lines were generated: Tg(Rho:EGFP)^+/+^, Tg(Rho:Prpf31:mCherry)^+/- ^and Tg(Rho:AD5:mCherry)^+/-^. A double transgenic line was generated by crossing Tg(Rho:EGFP)^+/+ ^and Tg(Rho:AD5:mCherry)^+/-^.

### Transient expression assay and retina imaging

Plasmids Rho:Prpf31:mCherry, Rho:AD5:mCherry Rho:SP117:mCherry, and Rho:mCherry were injected into 1-cell stage Tg(Rho:EGFP)^+/+ ^embryos, respectively. Larvae with abundant mCherry and EGFP expression were sorted and analyzed at 7 dpf. Fish larvae were fixed in 4% paraformaldehyde (PFA) in PBST and sectioned with Cryostat (Leica CM1850). Z-stack images of the fish eye sections were taken with a LSM510 Meta confocal microscope (Zeiss).

### Western Blot

Ten embryos each were homogenized in 100 μl lysate buffer (10 mM Tris/HCl pH6.8; 10% glycerol; 1% SDS; 25 mM β-mercaptoethanol; 0.005% bromophenol blue). 15 μl lysate each were analyzed on 12% SDS-polyacrylamide gels and transferred to nitrocellulose membrane. Membranes were blocked with 5% non-fat dried milk in 0.5% Tween-20/TBS for 1 hour at room temperature. Anti-PRPF31 primary antibody was used at a 1:250 dilution (generously provided by Bastian Linder). HRP-conjugated secondary antibody was used at a 1:10,000 dilution and protein bands were visualized by ECL (Pierce). Intensity of protein bands was analyzed using ImageJ [56]. Western blots were done in triplicates and data were assessed using student's t-test. Significance was defined as a P-value of less than 0.05 (95% confidence).

### qRT-PCR

RNA was extracted from zebrafish embryos or adult fish eyes. cDNA was prepared as described above and used as template for qRT-PCR. A negative control was prepared without adding transcriptase. Primers were designed to generate a 50-300 bp PCR product and are listed in Additional file 6, Table S2. For qRT-PCR, 1 μl of cDNA was used in a 25 μL reaction (12.5 μl qPCR MasterMix (Fermentas); 0.05 μl of ROX (Fermentas); 1 μl of each PCR primer (First base), and 9.45 μl of sterile water). An Applied Biosystem ABI Prism 7000 Sequence Detection System was used with the following conditions (10 min at 95°C and 40 cycles of 95°C for 15 s, 60°C for 1 min). The rhodamine derivate ROX present in the PCR master mix was used as a passive reference to normalize the signal. Negative template control and one additional no template control were included with each PCR run. Threshold cycle (Ct) values were automatically estimated. Relative quantification was done by using β-actin as internal control. Three to five biological and three technical repeats were conducted to acquire data that were analyzed using the ABI Prism 7000 SDS Software. All data are shown as means ± SEMs. Differences between groups were assessed using T-test with GraphPad Prism online software http://www.graphpad.com/quickcalcs/ttest1.cfm. Significance was defined as a *P*-value of less than 0.05 (95% confidence).

### Immunohistochemistry

Fish larvae and adult fish eye were fixed in 4% paraformaldehyde (PFA) in PBST overnight at 4°C. Samples were washed by PBST twice and soaked in 30% sucrose/PBST solution overnight at 4°C. Samples were then embedded in O.C.T. medium (Leica) and rapidly frozen in liquid nitrogen. 10 μm sections were cut at -22°C and adhered onto frosted slides (Fisher Scientific). After drying at 37°C for 30 min, sections were rehydrated with PBST at room temperature for 5 min. 1% BSA/PBST solution was applied onto the slides for 1 hour at room temperature for blocking. Sections were incubated with primary antibody anti-PRPF31 diluted in 0.1% BSA/PBST (1:100 dilution) overnight at 4°C. After 6 times PBST washing (10 min each), sections were incubated with secondary antibody AlexaFluor 488-conjugated anti-rabbit antibody (Invitrogen) for 1 hour at room temperature. After 5 times PBST washing, nuclei were stained with DAPI. Samples were mounted in Mowiol and images were acquired with a LSM510 Meta confocal microscope (Zeiss).

### Detection of cell death

Apoptotic cells were detected by TUNEL assay (Millipore) on cryosections. Adult retina sections were washed with PBST and equilibrated. TdT reaction mix was applied for 60 min at 37°C and the reaction was stopped with washing buffer. After washing in PBS three times (1 min each), specimens were incubated in 1% BSA/PBST solution for 1 hour at room temperature for blocking. Mouse monoclonal anti-DIG antibody and AlexaFluor 633-conjugated secondary antibody were used at 1:100 dilutions. After staining with DAPI, sections were mounted in Mowiol and images were taken with a LSM510 confocal microscope (Zeiss). TUNEL positive cell numbers were counted in 3 sections through optic nerve and 3 sections behind optic nerve from each retina. Statistic analysis was done in 5 fish retinas by using T-test.

## Competing interests

The authors declare that they have no competing interests.

## Authors' contributions

JY and CW conceived and designed the study. JY collected and assembled the data. JB participated in experiments and contributed to data analysis. JY and CW wrote the manuscript. UF contributed to project design and manuscript preparation. All authors read and approved the final manuscript.

## Supplementary Material

Additional file 1 **Figure S1. Alignment of human and zebrafish PRPF31 amino acid sequences**. Conserved NOSIC and NOP domains as well as predicted nuclear localization signals (NLS) are indicated with red, green and blue boxes, respectively. Frameshift positions caused by AD5 and SP117 mutations are indicated by vertical lines. The construct for expression of the SP117 mutant was generated by site-specific PCR mutagenesis using two completely matching primers (A and B) designed with a 1 bp insertion between 801 bp and 802 bp of p*rpf31*. The 5' region of the SP117 mutant was amplified using a 5' end out primer and reverse primer A at insertion area. The 3' region was amplified by a 3' end out primer and forward primer B at insertion area. 5' and 3' end PCR products have a 35 bp overlapping sequence derived from the matched primers, which can be annealed and extended in the third round of PCR. Full length SP117 mutant was amplified in the third PCR round with two outer primers and with a 5' and 3' end PCR product mix as template. All three fragments were subcloned into pCS2+ vector using BamHI and XhoI restriction sites. Click here for file

Additional file 2 **Figure S2. RNA levels after injection of 61 nM *prpf31*, AD5, SP117 mRNAs into zebrafish embryos by qRT-PCR**. At 8 hour after injection, AD5 RNA level shows more significant reduction compared to *prpf31 *and SP117. Data were analyzed using T-test. Significant difference is indicated by asterisk. Click here for file

Additional file 3 **Figure S3. TUNEL assay in AD5 and Prpf31 transgenic fish at 21 dpf**. (A) TUNEL assay in double transgenic fish expressing Rho:AD5:mCherry and Rho:EGFP. (B) TUNEL assay in double transgenic fish expressing Rho:Prpf31:mCherry and Rho:EGFP. (C) TUNEL assay in control transgenic fish expressing Rho:EGFP. Arrows indicate apoptotic cells detected and counted in the rod nuclei layers (RNL). CNL, cone nuclear layer; RNL, rod nuclear layer; INL; inner nuclear layer. Asterisks in A mark unspecific fluorescence in lens due to reflections. Click here for file

Additional file 4 **Figure S4. Detection of AD5:mCherry and Prpf 31 protein in AD5 transgenic fish retina by Western blot**. In the adult Tg(Rho:AD5:mCherry) fish retina, AD5:mCherry is expressed at significantly lower levels than endogenous Prpf31. Click here for file

Additional file 5 **Table S1. Primers used for cloning**. A list of primers used for cloning the described DNA constructs. All sequences are given in 5' to 3' orientation. Click here for file

Additional file 6 **Table S2. Primers used for qRT-PCR**. A list of primers used for real-time qRT-PCR. All sequences are given in 5' to 3' orientation. Click here for file
