# Supplementary figures and images for: Mutant Prpf31 causes pre-mRNA splicing defects and rod photoreceptor cell degeneration in a zebrafish model for Retinitis pigmentosa
Source: Mol Neurodegener. 2011 Jul 30;6:56. doi: 10.1186/1750-1326-6-56 (PMC3158551; doi:10.1186/1750-1326-6-56)

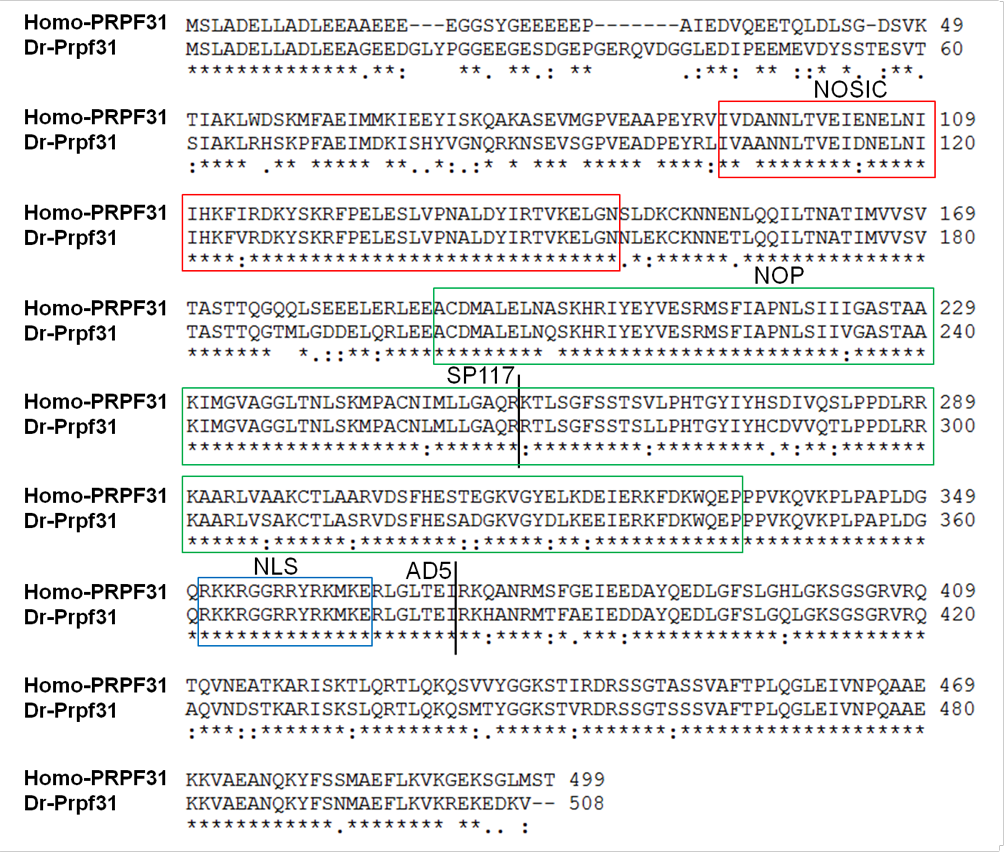

Supplement: Additional file 1 — Figure S1. Alignment of human and zebrafish PRPF31 amino acid sequences. Conserved NOSIC and NOP domains as well as predicted nuclear localization signals (NLS) are indicated with red, green and blue boxes, respectively. Frameshift positions caused by AD5 and SP117 mutations are indicated by vertical lines. The construct for expression of the SP117 mutant was generated by site-specific PCR mutagenesis using two completely matching primers (A and B) designed with a 1 bp insertion between 801 bp and 802 bp of prpf31. The 5' region of the SP117 mutant was amplified using a 5' end out primer and reverse primer A at insertion area. The 3' region was amplified by a 3' end out primer and forward primer B at insertion area. 5' and 3' end PCR products have a 35 bp overlapping sequence derived from the matched primers, which can be annealed and extended in the third round of PCR. Full length SP117 mutant was amplified in the third PCR round with two outer primers and with a 5' and 3' end PCR product mix as template. All three fragments were subcloned into pCS2+ vector using BamHI and XhoI restriction sites. [file 1750-1326-6-56-S1.TIFF]

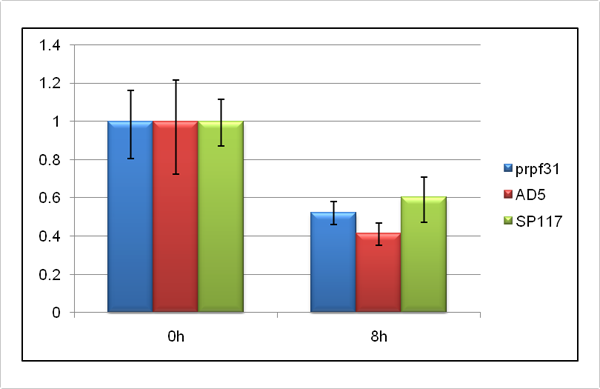

Supplement: Additional file 2 — Figure S2. RNA levels after injection of 61 nM prpf31, AD5, SP117 mRNAs into zebrafish embryos by qRT-PCR. At 8 hour after injection, AD5 RNA level shows more significant reduction compared to prpf31 and SP117. Data were analyzed using T-test. Significant difference is indicated by asterisk. [file 1750-1326-6-56-S2.TIFF]

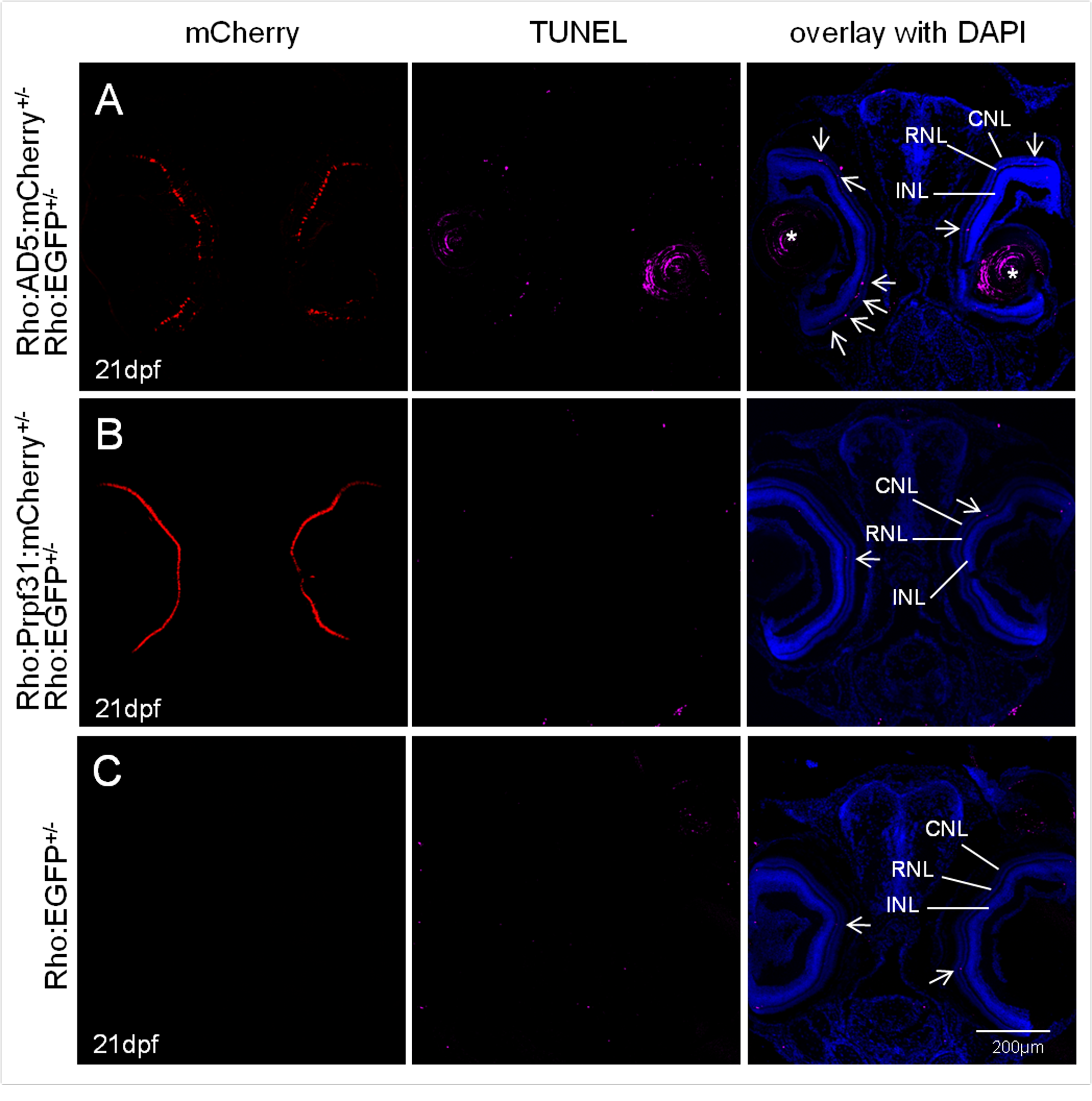

Supplement: Additional file 3 — Figure S3. TUNEL assay in AD5 and Prpf31 transgenic fish at 21 dpf. (A) TUNEL assay in double transgenic fish expressing Rho:AD5:mCherry and Rho:EGFP. (B) TUNEL assay in double transgenic fish expressing Rho:Prpf31:mCherry and Rho:EGFP. (C) TUNEL assay in control transgenic fish expressing Rho:EGFP. Arrows indicate apoptotic cells detected and counted in the rod nuclei layers (RNL). CNL, cone nuclear layer; RNL, rod nuclear layer; INL; inner nuclear layer. Asterisks in A mark unspecific fluorescence in lens due to reflections. [file 1750-1326-6-56-S3.TIFF]

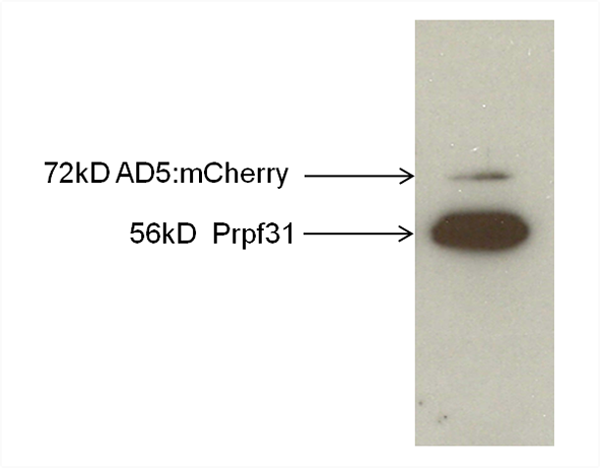

Supplement: Additional file 4 — Figure S4. Detection of AD5:mCherry and Prpf 31 protein in AD5 transgenic fish retina by Western blot. In the adult Tg(Rho:AD5:mCherry) fish retina, AD5:mCherry is expressed at significantly lower levels than endogenous Prpf31. [file 1750-1326-6-56-S4.TIFF]
