# Supplementary material for: Mutant Prpf31 causes pre-mRNA splicing defects and rod photoreceptor cell degeneration in a zebrafish model for Retinitis pigmentosa
Source: Mol Neurodegener. 2011 Jul 30;6:56. doi: 10.1186/1750-1326-6-56 (PMC3158551; doi:10.1186/1750-1326-6-56)
Supplement: Additional file 5 — Table S1. Primers used for cloning. A list of primers used for cloning the described DNA constructs. All sequences are given in 5' to 3' orientation. [file 1750-1326-6-56-S5.DOC]

**Additional file 5, Table S1.** Primers used for cloning.

| **PCR product** | **5’ primer** | **3’ primer** |
| --- | --- | --- |
| *prpf31* | GGATCCATGTCTTTGGCAGACGAGCT | CTCGAGTCAGACCTTGTCCTCCTTC |
| AD5 | GGATCCATGTCTTTGGCAGACGAGCT | CTCGAGTTAGATCTCAGTCAGTCCCAGTC |
| SP117 5’ product | GGATCCATGTCTTTGGCAGACGAGCT | GAATCCAGACAGTGTTCTTCCTCTGCGCTCCCAGC (primerA) |
| SP117 3’ product | GCTGGGAGCGCAGAGGAAGAACACT  GTCTGGATTC (primer B) | CTCGAGTTAGATCTCAGTCAGTCCCAGTC |
| SP117 | GGATCCATGTCTTTGGCAGACGAGCT | CTCGAGTTAGATCTCAGTCAGTCCCAGTC |
| *prpf31* MO | TGTCTTTGGCAGACGAGCTGCTTG | |
| *prpf31*-rescue | GGATCCATGTCCTTAGCTGATGAACT  CCTA | CTCGAGTCAGACCTTGTCCTCCTTC |
| AD-rescue | GGATCCATGTCCTTAGCTGATGAACT  CCTA | CTCGAGTTAGATCTCAGTCAGTCCCAGTC |
| SP117-rescue | GGATCCATGTCCTTAGCTGATGAACT  CCTA | CTCGAGTCACAGTGGTAGATGTAGCCT |
| Rho-promoter | GTGCTTCAATCAGATGCGGTG | GGCTGCGGTTGGATGTGGC |
| Prpf31-transgene | GGATCCATGTCTTTGGCAGACGAGCT | AAGCTTGACCTTGTCCTCCTTCTC |
| AD5-transgene | GGATCCATGTCTTTGGCAGACGAGCT | AAGCTTGATCTCAGTCAGTCCCAG |
| SP117-transgene | GGATCCATGTCTTTGGCAGACGAGCT | AAGCTTCAGTGGTAGATGTAGCCT |
| EGFP | GAATTCATGGTGAGCAAGGGCGAG | TGCTCAGGTAGTGGTTGTCG |
| mCherry | GAATTCGGATCCAAGCTTATGGTGAGCAAGGGCGAG | TCTAGATTACTTGTACAGCTCGTCCA |
