# Supplementary material for: Mutant Prpf31 causes pre-mRNA splicing defects and rod photoreceptor cell degeneration in a zebrafish model for Retinitis pigmentosa
Source: Mol Neurodegener. 2011 Jul 30;6:56. doi: 10.1186/1750-1326-6-56 (PMC3158551; doi:10.1186/1750-1326-6-56)
Supplement: Additional file 6 — Table S2. Primers used for qRT-PCR. A list of primers used for real-time qRT-PCR. All sequences are given in 5' to 3' orientation. [file 1750-1326-6-56-S6.DOC]

**Additional file 6, Table S2.** Primers used for qRT-PCR.

| **PCR product** | **5’end primer** | **3’ end primer** |
| --- | --- | --- |
| *prpf31* (endogenous) | GTCGTTGCATGTTGGCTTCTG | TGCTGTAATCCACCTCCATCT |
| *prpf31* (exogenous) | GAATACAAGCTACTTGTTCTTTTTGC | TGCTGTAATCCACCTCCATCT |
| *egfp* | ACGTAAACGGCCACAAGTTC | ACGTAGCCTTCGGGCATG |
| *rho* | ACCCCTCAACTACATCCT | GACCATAGCCCCATCTCA |
| *gnat1* | TGTCTATGTGAGGTGGATGACTG | AGCTTGCTAACTGTGCTCCTG |
| *gnat2* | AACAACCTCCACATCCCAAC | GCTTCTCTACAAGCGCCATT |
| *guk1* | TTCACGCACAGTAAGGACTTGT | GATACCACACAGTTTCCCCACT |
| *rcv1* | GGCCATATGCTTCCCACTT | GGGTCCATTTCAGCACCTTA |
| *calb2* | GCATGCCTTTCCCAAAGATA | AGCAGAGACTGCACGCTGTA |
| *crx* | GGAAGGAAAGATGCGTGAAG | TCACAATGTCTCCGGCAATA |
| *rx1* | GGGACAGAAACGATTAGTCTGG | GATAGTTTCCCCTTCTGCTCCT |
| *rx3* | CAGTGGTCTGGATCCGTTTT | TCCTAACTCCCTCTTCAACTGC |
| *gnat1*-intron5 | TACACCACAGCAAACCGAAA | CACTCAGAGCAGCGATGAAG |
| *gnat1*-intron7 | TCTGCAACCACCGTTACTTC | AAACGCTGTGTTTTCAGGAGA |
| *gnat1*-intron8 | CAGTAGAAATCGGCTGAATGG | CGAGCTACAAGGAGCAAAGC |
| *crx*-intron1 | GCCCACGTTCAAACAGAAAT | TCCAAACTTAATGCTCGTGCT |
| *crx*-intron2 | GAAATAAGCCAGCCATTTGGT | GCATTTAGCACGACGGTTCT |
| *rx3*-intron1 | TCTGAACGCGTTGACAACTT | CTAAACACACGTGCATAAAACGTAG |
| *rx3*-intron2 | TCCTCCACTTGTTCCAAACC | GCTGGGTTATTCCAGCATTC |
| *calb2*-intron8 | GAACCTGATGGCAGATTGGT | AGTGAATGTGGGGAGACTGG |
| *calb2*-intron9 | GCTATACCGCATGTCTTTGGA | GCTGTTGGGGGATATGAGAA |
| *calb2*-intron10 | TTTCGTGCTTGCTGTCAATC | TCTGACTCCAAGGGCAAATC |
| *rx1*-intron1 | ATTTGCGGGGAATCTTCTCT | CAACTTGATGTCTGTTGGAGGA |
| *rx1*-intron2 | TTTGGCCTGGAGTATTACGG | GGCGACTGCATGTAAACTCA |
| *rx1*-intron3 | GCCAAGACGCACAGTTGTAA | TCTGCGATTCTGGAACCAA |
| *gnat2*-intron1 | ACGGTCAAACTTCTGCTGCT | GCAAAGTAGTTGCTGGCACA |
| *gnat2*-intron3 | CGCATTCAGAGACCAGTTGA | CATATCTCTCCTTGCTAGCTCCTC |
| *gnat2*-intron5 | CTACTTGAACGAAATGGAC | CCAACTATTGCTTCCATC |
| *gnat2*-intron7 | GAGCATCTGCTTCCCTGACTAT | GGTCTGGATTGAACTGGAAGAG |
| *gnat2*-intron8 | AGTGATCCCAGATGAATAGC | TTCACCAGAGTAAATCCAAT |
